# Online ethics: where will the interface of mental health and the internet lead us?

**DOI:** 10.1186/s40345-017-0095-3

**Published:** 2017-08-06

**Authors:** Victoria Cosgrove, Emma Gliddon, Lesley Berk, David Grimm, Sue Lauder, Seetal Dodd, Michael Berk, Trisha Suppes

**Affiliations:** 1 Department of Psychiatry and Behavioral Sciences, Stanford University School of Medicine, 401 Quarry Road, Stanford, CA 94305 USA; 2 Bipolar and Depression Research Program, VA Palo Alto Health Care System, Palo Alto, USA; 3 IMPACT Strategic Research Centre, Deakin University, Geelong, Australia; 4 Department of Psychiatry, University of Melbourne, Parkville, Australia; 5 School of Population Health, University of Melbourne, Parkville, Australia; 6 School of Health Sciences and Psychology, Faculty of Health, Federation University Australia, Ballarat, Australia; 7 University Hospital Geelong, Barwon Health, Geelong, Australia; 8 Orygen, the National Centre of Excellence in Youth Mental Health, Melbourne, Australia; 9 Florey Institute for Neuroscience and Mental Health, Melbourne, Australia

## Abstract

While e-health initiatives are poised to revolutionize delivery and access to mental health care, conducting clinical research online involves specific contextual and ethical considerations. Face-to-face psychosocial interventions can at times entail risk and have adverse psychoactive effects, something true for online mental health programs too. Risks associated with and specific to internet psychosocial interventions include potential breaches of confidentiality related to online communications (such as unencrypted email), data privacy and security, risks of self-selection and self-diagnosis as well as the shortcomings of receiving psychoeducation and treatment at distance from an impersonal website. Such ethical issues need to be recognized and proactively managed in website and study design as well as treatment implementation. In order for online interventions to succeed, risks and expectations of all involved need to be carefully considered with a focus on ethical integrity.

## Background

E-health initiatives are poised to revolutionize delivery and access to mental health care around the world. For example, available applications focusing on assessment or intervention for adults with depression or anxiety (Christensen et al. 2014; Griffiths et al. 2010) embody considerable strengths such as global accessibility, reduced cost, consumer interactivity, and potential for personalization (Lal and Adair 2014). While moving mental health assessment and intervention online transforms the landscape of service provision, it also warrants ethical considerations that differ from those in traditional, in-office, face-to-face sessions. Conducting clinical research within online platforms delivering mental health care often necessitates an even more careful approach to ensuring ethical principles are upheld. While it can be argued that delivery online involves a similar ethical framework to face-to-face, the nuance of this is quite different in the online milieu.

This paper examines the unique challenges and ethics of overseeing a global, online clinical trial of a self-help intervention for individuals with bipolar disorder, MoodSwings 2.0. In clinical research studies with high-risk populations, protecting patients and minimizing their clinical risk are paramount to most other concerns. While this remains true for online mental health programs, the conceptualization of risk must be broadened to include potential breaches of confidentiality related to online communications (such as unencrypted email), data privacy and security, risks of self-selection and diagnosis as well as the shortcomings of receiving psychoeducation and treatment at distance, from an impersonal website rather than a human being. It is now recognized that face-to-face psychosocial interventions, designed to have beneficial psychoactive effects, can at times inadvertently have adverse psychoactive effects. This paper will present our views on the unique ethical challenges presented by the MoodSwings 2.0 online program for bipolar disorder, the lessons learned during our clinical trial, and potential future ethical considerations of online psychotherapeutic research.

### Mood and the internet

A 2014 landmark study, coauthored by representatives from the Core Data Science Team at Facebook as well as academicians at Cornell and Princeton, showed convincing experimental evidence that users’ moods, measured by tendencies to post positive or negative sentiment, could at least in part be altered by manipulating the type and amount of emotional content in a user’s Facebook “News Feed” (Kramer et al. 2014). In other words, website content may cogently shift and transform mood states independent of in-person meetings and exchanges. Simply, Facebook impacts mood.

So if online websites like Facebook that lack explicit mood-related messages or tools still have the capacity to shift mood, online mental health programs targeted to clinical populations like MoodSwings 2.0 with unequivocal psychoeducation and techniques specifically designed to facilitate mood management may be uniquely positioned to do so. The MoodSwings 2.0 program is an internet-based psychoeducational and supportive intervention for individuals with bipolar disorder, which is a chronic and disabling condition associated with frequent relapse and subsyndromal symptoms between episodes of mania, hypomania, or depression as well as significant impairments in occupational and social functioning (Judd et al. 2008; Perlis et al. 2006). Its self-guided design complements and serves as an adjunct to clinical care from local psychiatrists, psychologists, and other mental health professionals. The program’s content is based on the successful face-to-face group therapy program known as MAPS, which was adapted for online use and became known as MoodSwings.

A previous head-to-head trial of the MoodSwings program had promising results (Lauder et al. 2015), leading to a technological upgrade and re-launch of the program now known as MoodSwings 2.0. The first trial compared a basic version of MoodSwings consisting of psychoeducational materials and asynchronous discussion boards with a more interactive version that added skills-based cognitive behavioral therapy tools. Both groups showed clinically significant baseline to endpoint reductions in symptoms of mania and depression as well as other improvements in quality of life and functionality, and the more interactive version was superior to the basic version on long-term improvement in mania symptoms at 12-month follow-up. Additionally, MoodSwings 2.0 integrated video-based content that enhanced didactic experiences for participants whenever possible as well as ensured that the overall program worked on a variety of different platforms including PCs, MacIntosh, Iphones, Ipads, and other tablets.

A number of internet-based programs have been evaluated as adjunctive treatments for bipolar disorder (Hidalgo-Mazzei et al. 2015; Faurholt-Jepsen et al. 2015). Many studies have shown promising results, including improvements in quality of life, symptom severity, social support, and medication adherence; however, further investigation is needed to establish the potential benefits of these programs through controlled trials as few programs have been evaluated against active control conditions, noting that waitlist controls risk inflating effect sizes.

**Fig. 1:**
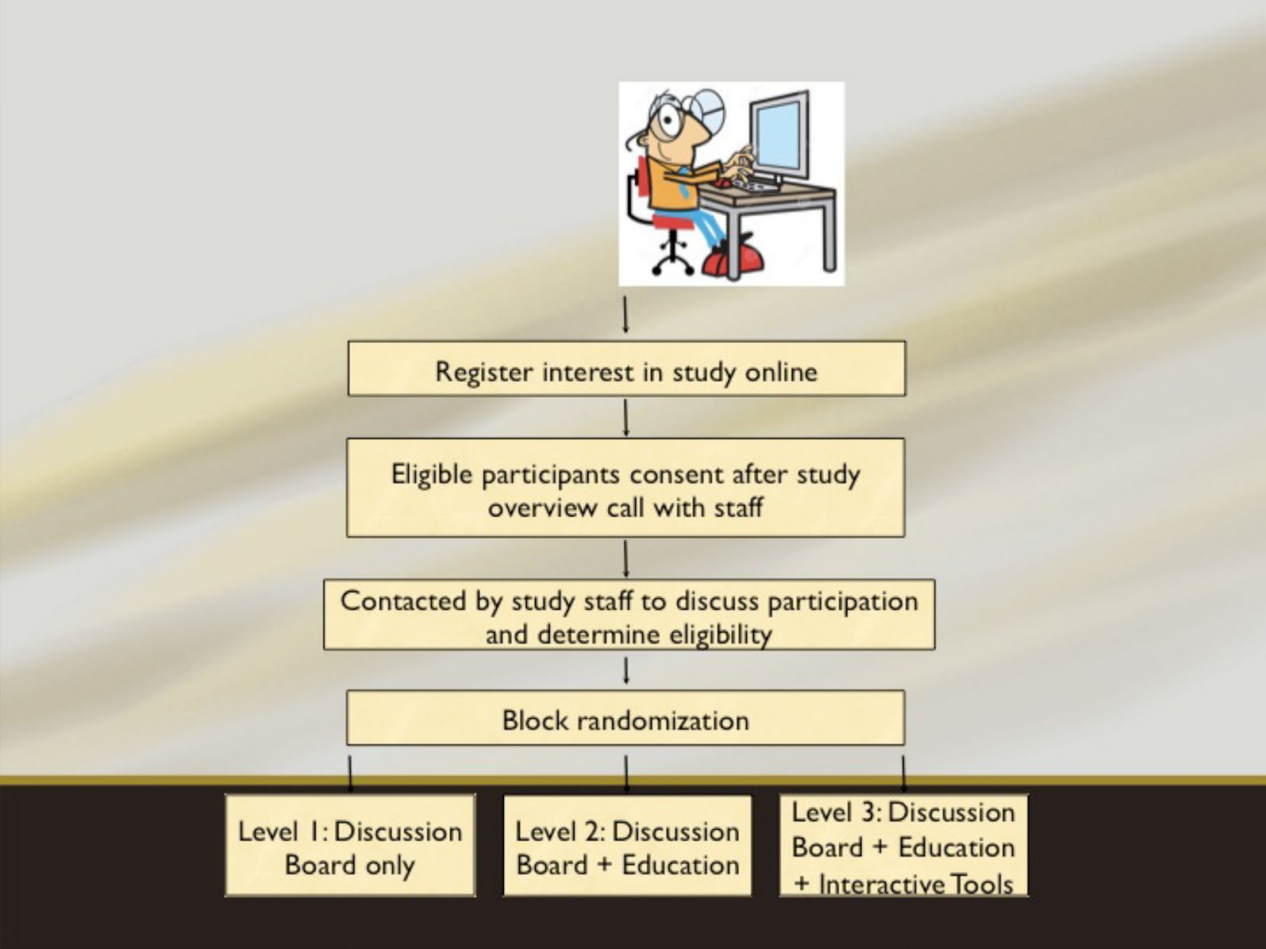
MoodSwings 2.0 study design. Figure shows flow for study procedures from first contact through intervention and follow-up

The MoodSwings 2.0 clinical trial, funded by the National Institute of Mental Health, is an international, randomized trial of three stepped levels of adjunctive treatment (Lauder et al. 2017) (see Fig. 1). Coordination of the clinical trial, which recruited three hundred participants globally, was managed via a two-site international collaboration between the Stanford University School of Medicine in California and Deakin University in Australia. Participants were subsequently assessed at 3, 6, 9, and 12 months via phone calls with trained study staff and online with standard instruments measuring mood symptoms, overall health status, and functioning, as well as quality of life in an effort to determine which components of care may be most valuable.

The potential benefits for participants in programs like MoodSwings 2.0 are considerable. Accessing specialty care and evidence-based clinical information for bipolar disorder often proves challenging for patients, particularly those who may live in rural areas or have minimal financial resources (Zeber et al. 2009). Consumers are often forced to seek in-person medication management from available medical doctors who may be excellent general practitioners but lack specialty training in the management of serious mental illnesses such as bipolar disorder. From the comfort of their own homes, MoodSwings participants were able to access psychoeducational materials vetted by worldwide experts in bipolar disorder. Additionally, individuals interacted on discussion boards, supported each other, and shared tips and techniques with other individuals from around the globe who struggle with similar mood symptomatology.

### The challenge of ensuring privacy

Human subjects research projects generally must receive approval from local ethics committees, such as institutional review boards (IRBs) in the United States or human research ethics committees (HRECs) in Australia (Harriman and Patel 2014). The Stanford Encyclopedia of Philosophy’s chapter on Internet Research Ethics indicates that privacy related to an individual’s confidentiality and anonymity as well as privacy and security of data is both exceptionally important when considering the ethics of conducting research on the internet (Buchanan and Zimmer 2013). Since the global internet is by definition a public forum, ensuring privacy is uniquely challenging for clinical researchers.

In MoodSwings 2.0, participant’s privacy and confidentiality were ensured in multiple ways. When a participant first registered his or her interest in the study at http://www.moodswings.net.au, he or she was asked to create a user name that did not resemble their own name or other names they may use on other websites such as discussion forums, Facebook, or Twitter. Previous experience from the MoodSwings 1.0 trial identified this possible issue, and this approach to prevent cross-site contamination was continued in MoodSwings 2.0. Further, participants and research staff communicated exclusively via an email messaging system that is internal to the website’s design. Participants were prohibited from using their personal email accounts for study communication and instead were always redirected to the internal messaging system. This maximized their privacy while enabling reliable and secure conversation between participants and study staff and is commonly used in online interventions (Klein et al. 2011).

Participants in MoodSwings 2.0 were able to communicate with other participants via one of three peer Discussion Boards, moderated by a researcher, depending on their randomization block. Here they were able to post interactive comments with other participants in the MoodSwings 2.0 study. While Board moderators were primarily on call to ensure patient safety (discussed below), maintaining privacy and anonymity was also considered when moderating posts. Moderators read posts with an eye toward editing out information that could potentially identify a participant, such as physical location (i.e., address).

Given that this trial was officially funded by two NIH grants separately awarded to one US and one Australian institution, formal engagement with two separate ethical review boards (e.g., IRB, HREC) was necessary. The two coordinating sites for the trial ensured that their local ethics boards were tasked with both adhering to high ethical standards for conducting research with humans as well as reviewing and approving the same amendments at the same time for the overall global project. However, given the online and global nature of the project, neither ethical entity was resourced to address legal issues that could arise specific to any geographic jurisdiction other than their own. Instead, the ethical review boards that vetted this clinical trial were only able to protect factors such as participant privacy and safety pertaining to their own local jurisdictions—in this case, California and Geelong (Australia).

A recent open pilot trial of a mindfulness-focused intervention for late-stage bipolar disorder modeled procuring ethical approval in one national jurisdiction (Swinburne University, Australia) while focusing study recruitment efforts in another (Canada) (Murray et al. 2015). This seems an ideal, honest, and transparent model for multi-national internet intervention research initiatives. Participants can then be clearly informed in consenting documents that the project in which they are considering participation has been ethically vetted by only one institution in a given geographic and legal jurisdiction. The challenge within a consent document seems to then become how to practically inform potential participants that regardless they are still themselves bound by the legal and ethical precedent in their own geographical jurisdiction.

### Does privacy equal security?

Of course, there are obvious ethical quandaries in confidently assuring patients of privacy when their research involvement occurs almost exclusively in a public setting like the internet. In some ways, as some bioethicists have suggested, the internet and other technologies may significantly decrease a patient’s sense of privacy (Weber et al. 2012). However, other studies suggest that the perceived potential to remain anonymous online may actually enhance a sense of privacy and safety (Griffiths et al. 2006). Findings from one study examining the safety, privacy, and security of an online treatment for young consumers recovering from early psychosis suggested that patients felt safe and trusted their experience on the website (Gleeson et al. 2014). Since MoodSwings 2.0 collected information about participants’ subjective experiences of their research participation, it is similarly well-positioned to explore subjective perceptions of privacy. It must be noted however that a sense of privacy does not always equate with actual security and protection of privacy.

In the United States, the Health Insurance Portability and Accountability Act of (1996) sets standards for Privacy and Security regarding Protected Health Information, or PHI. Privacy and security of data in clinical research is always a high priority. Since MoodSwings 2.0 operates on a virtual platform and collects data via the internet, careful considerations were made to protect and secure the data during negotiations with website developers. The MoodSwings 2.0 program adopts many common website security mechanisms including enterprise-based database encryption, Transport Layer Security (TLS)/Secure Sockets Layer (SSL) as well as disaster recovery plans (Baker and Bufka 2011). MoodSwings 2.0 also runs and is backed up on the Digital Ocean Solid State Drive Cloud Server, similar to cloud servers utilized by large-scale websites such as Pinterest and Facebook.

### Dr. Google

Appropriate utilization of online resources depends in part on a suitable match between the resource and the participant’s problem. A challenge for online resources is that of self-diagnosis and self-selection for treatment. Bipolar disorder is notoriously complex to diagnose and has many differential diagnoses. A considerable amount of diagnostic instability characterizes all psychiatric diagnoses. And “externalizing” diagnoses such as bipolar disorder may be more attractive to people than some “internalizing” diagnoses such as personality disorders. All of this is amplified by internet resources, which rely to a far greater extent on self-diagnosis and self-selection than face-to-face therapy and research. This entails a degree of risk and a corresponding ethical issue that will be a challenge for the field to resolve. MoodSwings 2.0 used telephone interviews as a part solution to this issue, but many online trial websites do not have the resources to do this, and this is not feasible if the promise of scale-independent roll out of such resources is to be realized.

### Safety Red Flags and the limits of internet interventions for mental health

**Fig. 2:**
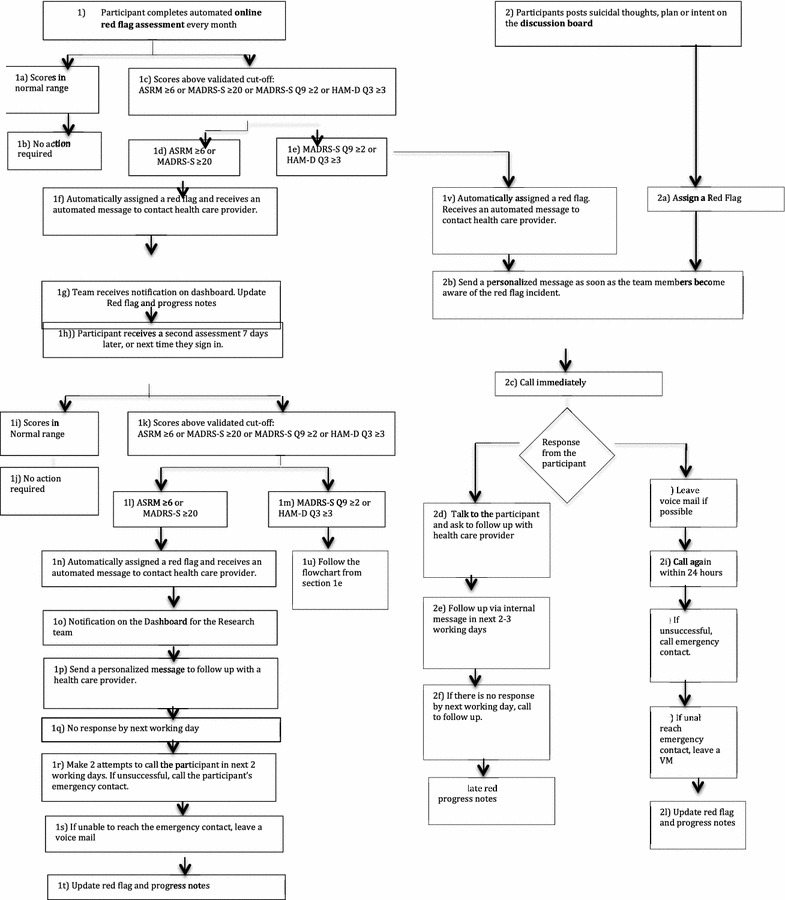
MoodSwings 2.0 Red Flag Monitoring System flowchart: self-assessment and discussion board components. Figure includes detailed model for Red Flag Monitoring System designed to identify via routine self-report methods study participants at high risk for suicidal behavior and provide guidance for services and care. Participants are also identified as high risk if the content of their discussion board posts is deemed concerning by study monitors

**Fig. 3:**
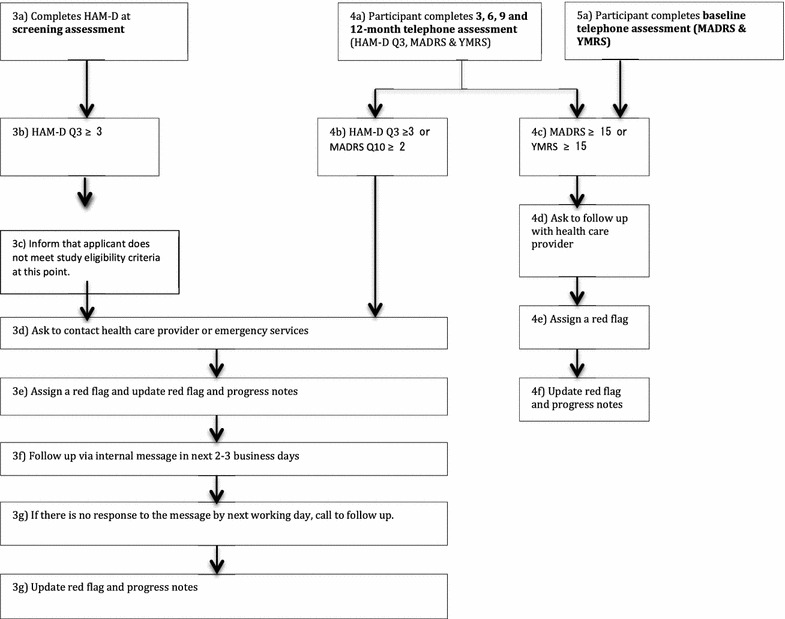
MoodSwings 2.0 Red Flag Monitoring System flowchart: interview components. Figure includes detailed model for Red Flag Monitoring System designed to identify via routine phone interview methods study participants at high risk for suicidal behavior and provide guidance for services and care

The MoodSwings 2.0 Red Flag Monitoring System (see Figs. 2, 3) was designed to identify and provide guidance to participants who may be approaching a clinical crisis. Although MoodSwings 2.0 was designed as an adjunct to local clinical care, a Red Flag System provides an ethical approach to fulfilling clinical responsibility to participants. When a participant signed consent and enrolled in the study, he or she was required to provide information for an individual who could be reliably contacted during an emergency. During regular online and phone assessments, there were two fundamental ways to receive a system “Red Flag.” First, scores above a validated cut-off on various self-report or interview-based, mood-related measures generated a Red Flag, sending an automated internal email with instructions to the participant to contact his or her health care provider. Most of these were the result of elevated overall scores on ratings of depression or mania, and in such cases study staff reassessed mood in 7 days.

However, some Red Flags were generated as a result of clearly expressed suicidal ideation or intent. In these cases, a member of the study team called the participant, and if unsuccessful at making contact, the emergency contact. A Discussion Board Moderator also had discretionary ability to generate a Red Flag for participants who expressed suicidal ideation, plan, or intent via a post. In this case, a participant was also contacted via phone.

The strengths and benefits of the MoodSwings 2.0 Red Flag Monitoring System included its benevolent objective to catch and help participants during clinical crisis by encouraging them to contact local care providers. Most of the time, the study team agreed that the Red Flag system was helpful for participants and researchers alike. However, there were noteworthy exceptions, where its implicit constraints should be underscored. For example, if study staff are not able to connect via phone for many days or at all with a participant or emergency contact, what steps should be taken to ensure patient safety? This is an ethical gray area connected to online interventions. For how long should study staff continue to attempt contact? What if a participant became agitated by the MoodSwings 2.0 automated email communication, which could occur as frequently as once per week if he or she is mid-episode?

The identification of participants who have been “flagged” as a result of general symptomatology or suicidal ideation also underscores the global nature of online internationally accessible projects as well as the limitations of practicing “distance therapy” (DeAngeles 2012). Participants in most cases lived in localities physically distant from Palo Alto or Geelong, the two study coordinating sites. Study staff were unlikely to be familiar with available crisis resources on a local level. Moreover, local resources were often scarce. During the course of the trial, the team sought to identify specific suicide hotlines or services for every country represented by active participants. For several remote countries where participants had enrolled, this proved impossible. This issue also underscored the difference between the quality and depth of the therapeutic linkages and relationships possible in online self-help forums and face-to-face trials, and the consequent feasibility of intervention in such diverse circumstances.

### Managing risk: protecting the interests of the MoodSwings 2.0 clinical research team

In addition to protecting patients, the interests of clinical researchers operating from a virtual and physical distance must be carefully considered. For example, there are inherent limitations when primary communication with participants takes place via electronic modalities such as email or discussion boards or phone-based clinical interviews. The most important safeguard for both the research team and participants was to emphasize repeatedly the adjunctive nature of the MoodSwings 2.0 Program. Participants as well as members of the study team were frequently reminded first and foremost that the MoodSwings 2.0 program was not a substitute for ongoing face-to-face supervision and consultation with health care providers. Study staff were prohibited from monitoring individual participant interaction with the MoodSwings 2.0 program or responding in personalized or individualized ways to participant input, except in cases of Red Flags.

As a further ethical check, a Data Safety Monitoring Board (DSMB) was convened specifically for MoodSwings 2.0, composed of ethicists and researchers from around the globe and with the sole purpose of identifying and managing specific risks for participants. DSMB discussions largely encompassed issues related to patient and data privacy and security described earlier in this opinion. DSMB members have also discussed the complexities of collecting information on adverse events (AEs) and serious adverse events (SAEs) frequently aggregated by researchers during clinical trials. Bioethicists are already uncertain about the quantity and type of AE and SAE information to solicit from participants during psychotherapy intervention trials (Czaja et al. 2006), and this question is even more complex within the context of a clinical trial of an online psychosocial intervention like MoodSwings 2.0. If AE and SAE data are systematically collected during patient assessments, can any be reliably attributed to passive interactions with online psychoeducational materials or discussion board posts, especially when levels of involvement with the website vary significantly from participant to participant? If a study interview reveals an AE or SAE that represents an ongoing, real-time crisis unrelated to bipolar disorder, what is the corresponding ethical responsibility of the interviewer? Should this information generate a Red Flag? From one perspective, it seems unethical to request and record information from participants about sensitive life events without any intent of clinical follow-up or planned utilization of the data. From another, it seems unethical not to probe.

From an ethical standpoint, it is also important to consider that internet interventions for mental health may not always prove helpful for their consumers. A recent analysis of negative effects from a pooled sample of participants in four separate internet-based cognitive behavioral therapy interventions for anxiety or depression found that 9.3% of participants reported at least one potentially treatment-related adverse event (Rozental et al. 2015). Subsequent qualitative content analysis suggested that gaining knowledge and awareness may have led to feeling anxious or depressed during treatment for some, while others indicated frustration and dysphoria related to persistent struggles implementing the content on the available internet platform. Even more salient are the recently published results from the MONARCA I trial, which focused on daily, electronic, self-monitoring in bipolar disorder using a randomized, placebo-controlled design (Faurholt-Jepsen et al. 2015). Interestingly, for participants receiving the intervention, depression scores were higher when compared with those of participants receiving a control condition. The authors suggest that the act of daily monitoring of symptoms of depression may have helped prolong them by continually drawing awareness and possibly increasing rumination (Berk and Parker 2009).

### Managing expectations

One unanticipated challenge encountered during the MoodSwings 2.0 trial involved managing the expectations of different entities including website developers, clinical researchers, and research participants from around the world, as these were sometimes at odds. Similarly, website developers functioning within a for-profit environment may not be sensitive to budget and personnel constraints faced by smaller-scale sites like MoodSwings 2.0 funded by not-for-profit sources. MoodSwings 2.0 participants are by definition individuals who actively engage in online activities. Such consumers of the internet often have expectations for the real-time availability and functionality of chosen websites. Participants may have more interactive familiarity with websites such as Facebook, Pinterest, or Twitter which have capital and scope to provide technologically sophisticated online experiences. A user expects to be able to access his Facebook news feed or Pinterest board on demand. Expectations for these websites are focused on their ability to provide immediate and uninterrupted entertainment. Further, a majority of internet consumers seek some kind of health information on the internet (Fox and Jones 2009), and their expectations are for sound information that they can use to inform health care decisions.

Individuals enrolled in an online program that provides treatment for mental illness, like MoodSwings 2.0 for bipolar disorder, likely possess slightly modified expectations for their virtual experience. An individual visiting a website like http://www.moodswings.net.au is likely in search of more than simple entertainment. It seems logical that participants have an ethical right to expect access to discriminating psychoeducation on bipolar disorder informed by the scientific evidence base as well as access to moderated and safe peer discussion forums.

In turn, the clinical research staff has the right to expect participants to safely and responsibly use the MoodSwings 2.0 program, which is designed to be an adjunct to in vivo clinical care. The ethical “catch” is that the research team has no way to globally enforce or verify that participants are seeking care on a local level. And sometimes an unexpected escalation of mood or psychotic symptoms during a bipolar episode may make it very difficult for a participant to physically reach out to a medical doctor or psychotherapist for immediate help. In some cases, participants instead opted to post to a MoodSwings 2.0 Discussion Board or send emails to research staff via the internal messaging system. Within the context of the trial, these instances generated Red Flags, and participants were contacted within days. It is vital that participants are informed and reminded about the limitations of entirely self-help online interventions and the link with their treating clinician is reinforced. Future online interventions need to be cognizant of moderating participant expectations and developing clear protocols to address patient distress.

## Conclusions

One commentary suggests that the “speed at which the internet can spawn new ethical dilemmas has thus far understandably outpaced the rate at which organized psychology can develop ethical principles in a careful, deliberative fashion” (Humphreys et al. 2000). The development of the MoodSwings 2.0 program and its accompanying trial has represented a collision of many different “worlds,” including proprietary software development companies, academics from the United States and Australia, clinical researchers accustomed to studies that enroll human subjects in-person, ethics committees with norms developed around face-to-face studies, and participants with serious mental illnesses from around the globe. In order for online interventions for mental health to succeed as either clinical trials or reputable clinical resources, risks and expectations of all involved need to be carefully considered with a focus on ethical integrity.
